# Improved brain functional network in major depressive disorder with suicidal ideation after individual target-transcranial magnetic stimulation treatment: a graph-theory analysis

**DOI:** 10.3389/fpsyt.2025.1486835

**Published:** 2025-01-22

**Authors:** Yao Zhang, Nan Mu, Shun Qi, Chuanzhu Sun, Yang Rao, Xinyi Yang, Jianying Guo, Yunfeng Mu

**Affiliations:** ^1^ Xijing Hospital, Air Force Medical University, Xi’an, Shaanxi, China; ^2^ The Key Laboratory of Biomedical Information Engineering of Ministry of Education, Institute of Health and Rehabilitation Science, School of Life Science and Technology, The Key Laboratory of Neuro-informatics & Rehabilitation Engineering of Ministry of Civil Affairs, Xi’an Jiaotong University, Xi’an, China; ^3^ Shaanxi Brain Modulation and Scientific Research Center, Xi’an, Shaanxi, China; ^4^ Department of Gynecological Oncology, Shaanxi Provincial Cancer Hospital, Xi’an, China

**Keywords:** depression, suicidal ideation, transcranial magnetic stimulation, small-world, graph-theory

## Abstract

**Introduction:**

Major depressive disorder with suicidal ideation (MDD/SI+) is characterized by high prevalence, high recurrence rate, high disability rate and low response rate. There is an urgent need for clarifying the pathogenesis and developing novel treatment methods.

**Methods:**

Subjects were recruited for the collection of Magnetic Resonance Imaging data and clinical scales. Individual target-transcranial magnetic stimulation (IT-TMS) using Stanford Neuromodulation Therapy over individualized left dorsolateral prefrontal cortex was performed to treat MDD/SI+ with the ethical approval (KY20212218-C-1). GRETNA software was used to analyze brain network characteristics according to graph theory.

**Results:**

A total of 32 patients (aged 18-55) and 28 healthy controls (aged 20-51) had been recruited. Patients after IT-TMS treatment had significant reduction in suicidal ideation and depressive symptom. The functional network of all three groups conformed to small-world topology. There was a renormalization in topology structure after IT-TMS treatment. Decreased functional connectivity between right insula and left anterior cingulate gyrus correlated with improvement in Beck Scale for Suicide Ideation scores.

**Discussion:**

The current study highlights that MDD/SI+ patients in this cohort showed abnormal brain network connections compared to healthy controls, and that IT-TMS may exert its treatment effects by reducing spontaneous hyper-connectivity in the salience network and insula.

## Introduction

Major depressive disorder (MDD) is the most common mental disorder, characterized by high prevalence, high recurrence rate, high disability rate and low response rate, with 58% MDD patients having suicidal ideation (MDD/SI+) and 15% committing suicide behavior (MDD/SB+) (1, 2). Although suicide prevention has been thoroughly researched, suicide remains a major cause of morbidity and mortality worldwide, causing a great burden to family and society (3). Only a few treatment options (e.g., lithium salt, antidepressant and cognitive therapy) are available, but these measures are also accompanied by side effects and unsatisfactory remission rate (4). Currently, the pathogenesis of MDD/SI+ is still not fully clear. There is an urgent need that clarifies the difference in brain functional networks underlying MDD/SI+ for developing novel treatment methods.

Brain functional networks are complex systems, and multiple connective networks serve different functions (5–7). For example, Chase et al. found the lower connectivity between the salience network and default mode network in MDD/SI+ patients compared with healthy volunteers (8). Kim et al. reported that the functional network connectivity between the left superior frontal gyrus and the rest brain regions were significantly reduced in MDD/SI+ patients (9). These results indicate that abnormality in the widespread functional network might contribute to the pathogenesis of MDD/SI+. Whereas, the functional changes of certain brain networks or regions cannot reflect the brain network mechanism overall. The graph-theory analysis from Magnetic Resonance Imaging (MRI) is to analyze the topology of the whole brain connectomes by calculating the global and local neural network features, which is more suitable for clarifying the potential brain functional differences (10). Brain networks derived from graph theory, including rich-club (11) and small-world (12) networks, are responsible for processing and delivering information efficiently. For example, decreased degree centrality value in a frontoparietal network was found in MDD/SI+ patients that could help distinguish patients from healthy individuals (13). Among the bipolar disorder patients with high risk of suicide, the dysfunction of hubs, including ventromedial prefrontal cortex and right anterior insula, was found using graph‐theory analysis (14). These hubs may be targets for novel therapeutics to reduce suicide risk in bipolar disorder. The connectomics-based functional network alterations in depressed patients with suicidal behavior were also reported (15). However, graph-theory analysis of whole brain functional connectivity in MDD/SI+ patients is still absent.

If delivered properly, transcranial magnetic stimulation (TMS) can modulate the excitability and plasticity of stimulated regions and other brain parts connected to them (16). Repetitive TMS (rTMS) has been used with varying degrees of effectiveness in various neurological and psychiatric diseases (17). For example, rTMS spans six consecutive weeks and meets approximately 32% remission and 49% response in major depression (18, 19). Another study targeted the left dorsolateral prefrontal cortex (DLPFC) and used rTMS to intervene in six suicidal ideation patients at a dose five times the FDA-approved standard dose, which is named Stanford Neuromodulation Therapy (SNT) (20). The results showed that after 5 days of treatment, the suicidal ideation score of the 6 patients decreased by 86.27% on average, indicating that rTMS is an efficient and safe intervention option for suicidal ideation. However, the mechanism by which TMS interferes with the brain functional connectivity of suicidal ideation is unknown.

In the current study, we applied SNT as an intervention option for MDD/SI+ patients combined with individual target-transcranial magnetic stimulation (IT-TMS), which represents an innovative tool that opens new avenues in the treatment of mental disorders, especially MDD (21). We used graph theory to analyze the functional separation and integration, verifying that functional network connectivity and efficiency will be improved after IT-TMS therapy.

## Materials and methods

### Ethical approval statement

Written informed consent was obtained from the individuals for the publication of any potentially identifiable images or data included in this article. This study was approved by the Research Ethics Review Board of the Institute of Mental Health of Xijing Hospital with the approval number: KY20212218-C-1 in 2021.

### Recruitment

Subjects were recruited from Xijing Hospital from January 2022 to December 2023. All MDD/SI+ patients were made a definite diagnosis using the Diagnostic and Statistical Manual of Mental Disorder. The inclusion criteria were as follows (1): right-handed (2); aged 18-60 (3); 17-item Hamilton Depression Rating Scale (HAMD-17) score >17 and Beck Scale for Suicide Ideation-Chinese Version (BSI-CV) score >6 (4); a negative urine drug screen, and a negative urine pregnancy test if female (5); acute suicide behavior (who needed immediate treatment) was excluded by clinical diagnosis and evaluation. The exclusion was: contraindications of TMS and MRI measurement. For safety, all MDD/SI+ patients were instructed to take venlafaxine or duloxetine constantly according to the doctor’s advice. The age- and gender-matched healthy controls (HCs) were also recruited.

### MRI data collection and processing

MRI data were acquired using a 3.0 T UNITED Discovery 770 MRI scanner (Shanghai, China). Subjects were required to keep still, think of nothing, and stay awake during the entire session. The resting-state functional images were obtained with the following parameters: field of view (FOV) = 224 × 224 mm, in-plane resolution = 64 × 64, echo time (TE) = 30 ms, repetition time (TR) = 2,000 ms, slice thickness = 4 mm, flip angle = 90^°^ and voxel size = 3.5 × 3.5 × 4 mm^3^. For anatomical reference, a high-resolution T1-weighted image was also acquired with the following parameters: TR = 7.24 ms, TE = 3.10 ms, FOV = 256 × 256 mm, flip angle = 10°, slice thickness = 0.5 mm and voxel size = 0.5 × 0.5 × 1 mm. MRI data were collected before the initiation of TMS treatment (baseline) and immediately after the last TMS treatment in patients’ group. MRI data of HCs were only collected at baseline.

MRI data were preprocessed using Data Processing and Analysis for Brain imaging (DPABI) software (http://rfmri.org/dpabi). The initial 10 volumes were discarded to avoid scanning noise. The remaining images were subjected to slice timing correction and motion realignment (less than 2 mm or 2°), during which the mean frame-wise displacement was calculated. Subjects with maximal translation more than 2 mm or maximal rotation more than 2° were excluded. Next, to regress out the nuisance signals from cerebrospinal fluid and white matter head motion effects, the Friston-24 model was used. The MRI data were finally normalized to the Montreal Neurological Institute (MNI) space using the diffeomorphic anatomical registration through exponentiated lie algebra (DARTEL) method, smoothed with a Gaussian kernel and band-pass filtered (0.01-0.08 Hz).

### IT-TMS treatment

The individualized TMS targets in left DLPFC (L-DLPFC) were calculated according to SNT algorithms (22–24). Firstly, each patient’s L-DLPFC and subgenual anterior cingulate cortex (sgACC) regions were subdivided into numerous functional subnuclei defined according to correlated voxel pairs using a hierarchical agglomerative clustering algorithm. For each subnucleus in L-DLPFC and sgACC, a single time-series value representing the single voxel time series that was most correlated with the median time series was identified. The correlation matrix between L-DLPFC and sgACC subnuclei was then calculated. Secondly, the optimal targets in L-DLPFC were determined by considering the most anticorrelation subnucleus, the larger size of subnucleus, and the higher spatial concentration, which are equally weighted in this algorithm.

IT-TMS was performed using the Black Dolphin Navigation Robot (S-50, a sub-millimeter smart positioning system, Solide Medical Co., Ltd., Xi’an, Shaanxi, China) with a figure-of-8 coil (YINGCHI, Shenzhen, China) by trained professionals. A 3D-printed mask with ball-like tracking points was attached to the patient’s head. The position of the tracking points and the location and orientation of the coil were co-registered and visualized by an infrared camera system, which allowed the precise and repetitive navigation of the coil over the target area under real-time visualization. Fifty intermittent theta-burst stimulation (iTBS) sessions (1,800 pulses per session, 50-min intervals) were delivered in 10 daily sessions over 5 consecutive days at a 90% resting motor threshold. These parameters were set according to SNT that was approved by FDA (25).

### Clinical assessment

Suicidal ideation was assessed using the BSI-CV and HAMD-17 before (baseline) and after IT-TMS therapy (follow-up: immediately, 15 days and 30 days after the last treatment). The assessment was done by the same trained physiotherapist. Response of suicidal ideation was defined as the reduction of more than 50% relative to the baseline BSI-CV score, and remission was defined as that BSI-CV score was 0 (22). Response of depression was defined as the reduction of more than 50% relative to the baseline HAMD-17 score, and remission was defined as that HAMD-17 score was less than 7.

### Brain network analysis

GRETNA software (http://www.nitrc.org/projects/gretna) was used to analyze brain network characteristics according to graph theory (11). Briefly, the whole brain was divided into 116 network nodes based on the automated anatomical labeling (AAL) atlas. Pearson correlation coefficient between the time series of all possible pairs of nodes was calculated, yielding a 116 × 116 correlation matrix for each subject, which was then transformed into an undirected binarized matrix using sparsity thresholding (5% ≤ s ≤ 40%) at an interval of 0.1. The functional segregation metrics consist of clustering coefficient (Cp), normalized clustering coefficient (γ), and local efficiency (Eloc). The functional integration metrics consist of characteristic path length (Lp), normalized characteristic path length (λ), and global efficiency (Eglob). The small-world metric includes σ (σ = γ/λ). These metrics were all obtained. The area under the curve (AUC) for each network metric was calculated for further statistical comparisons.

### Statistical analysis

SPSS software (version 26) was used for statistical analysis. Differences in demographic information (HCs vs. patients at baseline) and clinical scales (HCs vs. patients at baseline) were compared using the chi-square test and Student’s *t*-test. Differences in clinical scales (patients at baseline vs. patients at follow-up) were compared using ANOVA for repeated measurement. The demographic and clinical data were expressed as mean ± Standard Deviation (SD). Two-sample *t*-test (HCs vs. patients at baseline) or paired *t*-test (patients at baseline vs. patients at follow-up) were used to identify changes in global network metrics. The AUC values of each network metric showing abnormal differences were extracted and drawn. A network-based statistic (NBS) approach and paired *t*-test were used to reveal any significant differences in Edges and Nodes of node network (patients at baseline vs. patients at follow-up). BrainNet software was then used to visualize the brain regions with statistical differences. *P* value in node network was calculated using MedCalc software. Pearson analysis was performed to examine the correlation between changes in network metrics and clinical scores. Multiple comparisons were corrected by false discovery rate (FDR) with a corrected significance level of *P* = 0.05.

## Results

### Subjects

**Figure 1:**
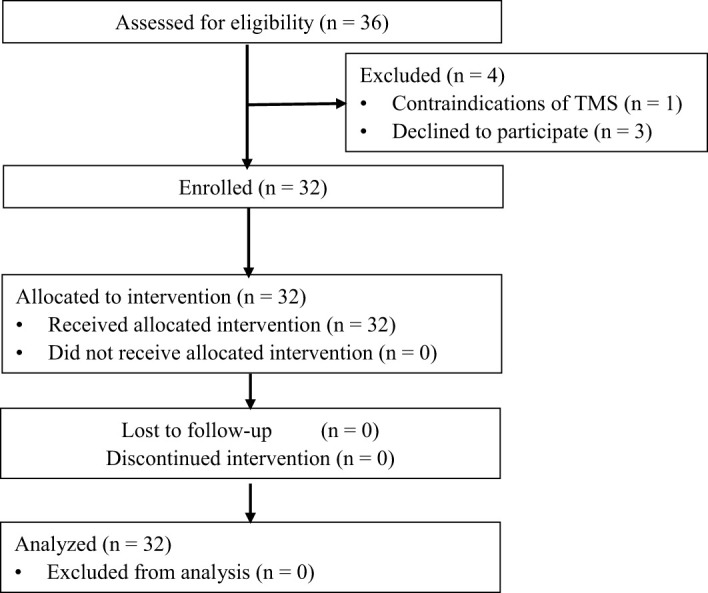
The STROBE flow diagram.

A total of 36 patients (aged 18-55) and 28 HCs (aged 20-51) had been recruited, among which 4 patients were excluded. All patients had suicidal ideation at the time of screening for BSI-CV (score > 6), and depression symptom for HAMD-17 (score > 17). There was no significant difference in age (*P* = 0.358), gender (*P* = 0.365), and year of education (*P* = 0.061) between patients and HCs (**Table 1**). The patients had significantly higher BSI-CV (17.6 ± 7.06, *P* = 0.000) and HAMD-17 (27.9 ± 4.31, *P* = 0.000) scores compared with HCs (**Table 1**). All patients were tolerable to TMS without dropping out. None of the HCs had psychopathy currently or previously. Detailed information is listed in **Table 1**. The STROBE flow diagram is shown below in **Figure 1**.

**Table 1:** Demographic and clinical characteristics of subjects at baseline.

Characteristics (mean (SD))	Patients (n=32)	Healthy controls (n=28)	*P* value
Age (years)	27.7 (10.38)	29.6 (5.01)	0.358
Gender (Male/Female)	5/27	7/21	0.365
Education (years)	13.34 (2.79)	15.8 (1.93)	0.061
BSI-CV	17.6 (7.06)	0.0 (0.00)	< 0.0001
HAMD-17	27.9 (4.31)	4.4 (2.60)	< 0.0001
Duration of illness (months)	11.4 (1.39)	–	–

At baseline, demographic and clinical characteristics were compared between patients and healthy controls.

P value: healthy controls vs. patients at baseline; SD, standard deviation; BSI-CV, Beck Scale for Suicide Ideation-Chinese Version; HAMD-17, 17-item Hamilton Depression Rating Scale.

As shown in **Table 2**, patients showed significant reduction in BSI-CV ((6.1 ± 5.98) < 6, *P* < 0.001) and HAMD-17 ((9.4 ± 5.43) < 17, *P* < 0.001) scores immediately after IT-TMS treatment. Moreover, the scores acquired 15 and 30 days after treatment continued the trend of decrease. The response and remission rates of suicidal ideation reached 93.75% and 37.50%, respectively, 30 days after treatment. There were no missing data in this study.

**Table 2:** Clinical characteristics of patients before and after IT-TMS.

Characteristics	Baseline (n=32)	Follow-up (n=32)											
		Immediately after IT-TMS treatment				15 days after IT-TMS treatment				30 days after IT-TMS treatment			
		Mean (SD)	*P*	Response (%)	Remission (%)	Mean (SD)	*P*	Response (%)	Remission (%)	Mean (SD)	*P*	Response (%)	Remission (%)
BSI-CV	17.6 (7.06)	6.1 (5.98)	< 0.0001	65.63	28.13	5.8 (6.01)	< 0.0001	75.00	28.13	3.2 (4.53)	< 0.0001	93.75	37.50
HAMD-17	27.9 (4.31)	9.4 (5.43)	< 0.0001	84.38	31.25	7.8 (4.57)	< 0.0001	93.75	50.00	5.5 (4.24)	< 0.0001	96.88	71.88

BSI-CV and HAMD-17 scores were compared before and after IT-TMS treatment.

P value: patients at baseline vs. patients at follow-up in BSI-CV or HAMD-17 scales; SD, standard deviation; IT-TMS, individual target-transcranial magnetic stimulation; BSI-CV, Beck Scale for Suicide Ideation-Chinese Version; HAMD-17, 17-item Hamilton Depression Rating Scale.

### Global network

**Figure 2:**
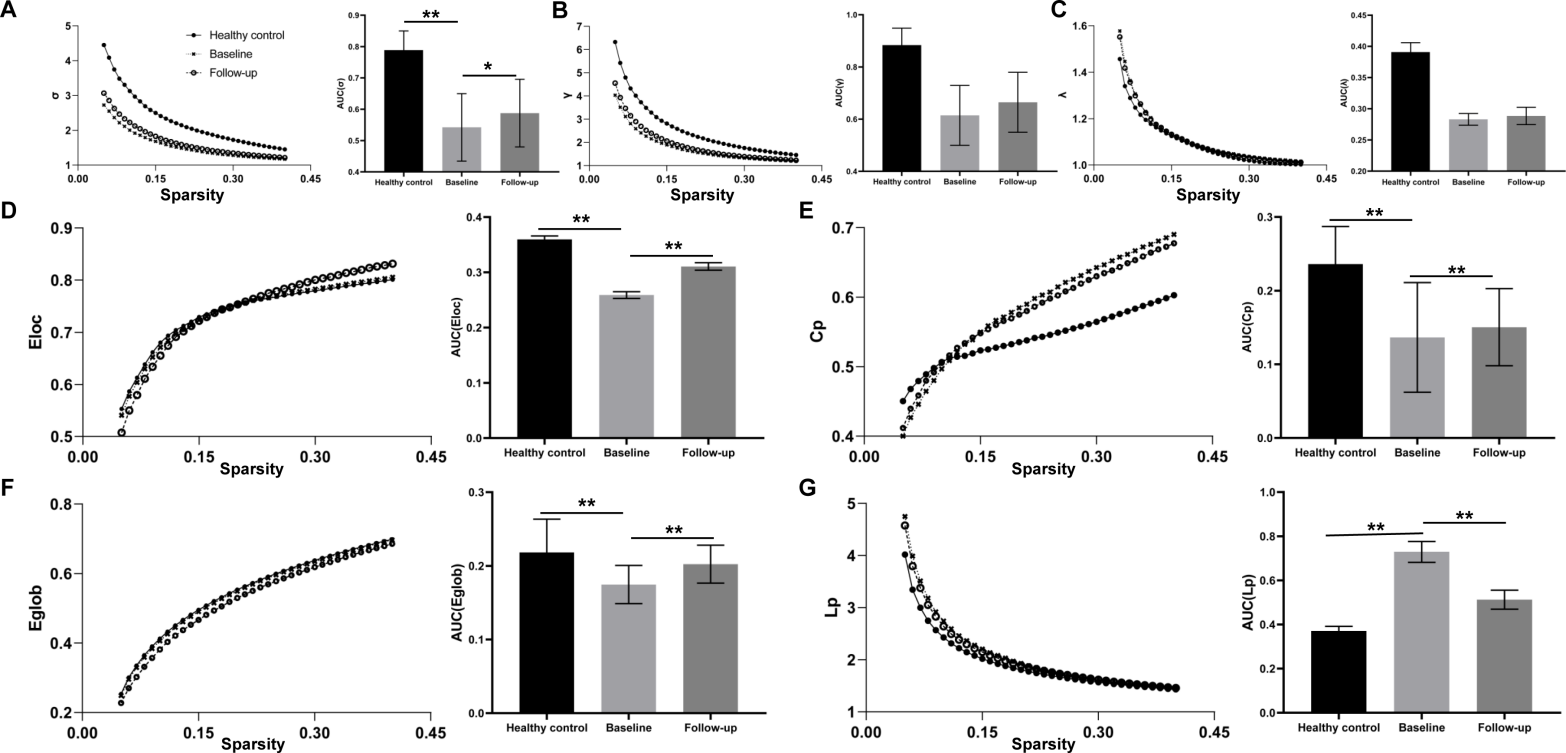
Alteration of global topological properties across the sparsity from 0.05 to 0.40, including the typical small-world network architectures (**A** σ > 1, **B**: γ > 1, and **C**: λ ≈ 1), the functional segregation metrics (**D**: Eloc and **E**: Cp) and the functional integration metrics (**F**: Eglob and **G**: Lp). The solid circle stands for the healthy control, the cross for the MDD/SI+ patients at baseline and the hollow circle for patients at follow-up. The area under the curve (AUC) for each topological property in healthy control at baseline, patients at baseline and patients at follow-up was calculated. *P<0.05. **P<0.01.

Overall, the functional networks of all three groups (HCs at baseline, patients at baseline and patients at follow-up) conformed to the small-world attribution (σ > 1, γ > 1, λ ≈ 1, **Figure 2**) using sparsity thresholding between 0.05 and 0.40. In addition, two-sample *t*-test results indicated that MDD/SI+ patients at baseline had altered global properties (decreased σ), reduced functional segregation metrics (Cp and Eloc), and abnormal functional integration metrics (increased Lp and decreased Eglob) compared with HCs at baseline. After IT-TMS treatment in the patients’ group, the above metrics at follow-up were significantly reversed compared with baseline (paired *t*-test, *P* < 0.05), indicating that the efficiency of functional separation and functional integration was improved.

### Node network

**Figure 3:**
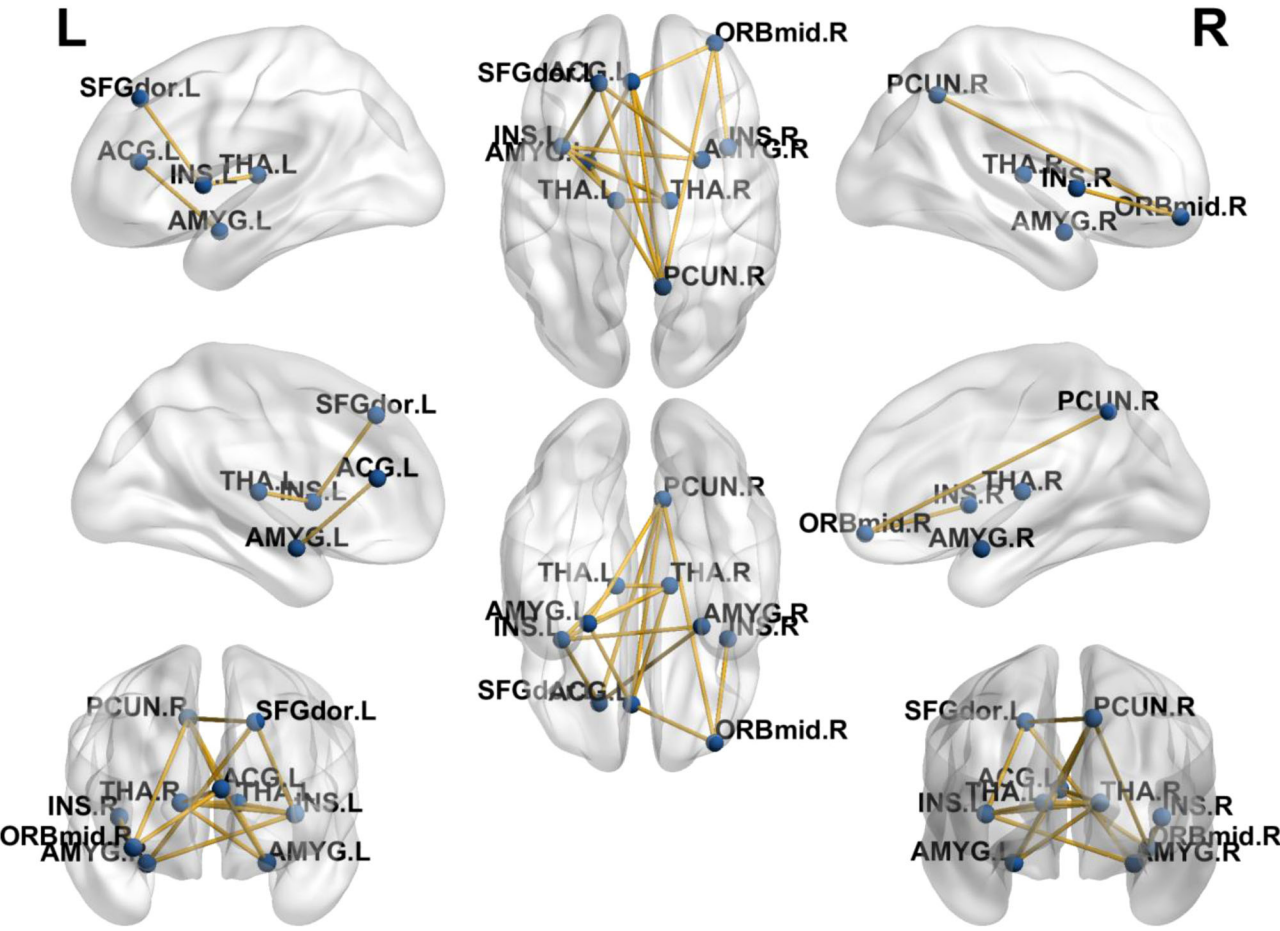
Alteration of functional connections of the node network after IT-TMS treatment in the patients. L, left; R, right. The nodes (blue points) and edges (yellow lines) are shown in this figure. The corresponding brain regions are also marked. The nodes were mainly located at superior frontal gyrus, anterior cingulate gyrus (ACG), insula, amygdala, thalamus and middle frontal gyrus (orbital part).

After NBS multiple correction and paired *t*-test (*P* < 0.05), the patients showed reduced functional connections of nodes at follow-up compared with baseline values. The nodes were mainly located at superior frontal gyrus, anterior cingulate gyrus (ACG), insula, amygdala, thalamus and middle frontal gyrus (orbital part) (**Table 3**). Detailed connections (Edges and Nodes) are shown in **Figure 3**.

**Table 3:** Node network with altered functional connections.

Regions	Betweenness (*P*)	Centrality (*P*)	Nodal efficiency (*P*)
baseline > follow-up			
Left			
Superior frontal gyrus (dorsolateral) (SFGdor)	0.0045	0.0012	0.0029
Insula (INS)	0.1038	0.0139	0.0289
Anterior cingulate gyrus (ACG)	0.0027	0.0148	0.0401
Amygdala (AMYG)	0.0010	0.0318	0.0180
Thalamus (THA)	0.0015	0.1469	0.1388
Right			
Precuneus (PCUN)	0.0008	0.0201	0.0378
Middle frontal gyrus (orbital part) (ORBmid)	0.0108	0.0358	0.1569
Insula (INS)	0.0098	0.0016	0.0018
Amygdala (AMYG)	0.0021	0.0250	0.0468
Thalamus (THA)	0.0016	0.0156	0.0038

### Correlation analysis

**Figure 4:**
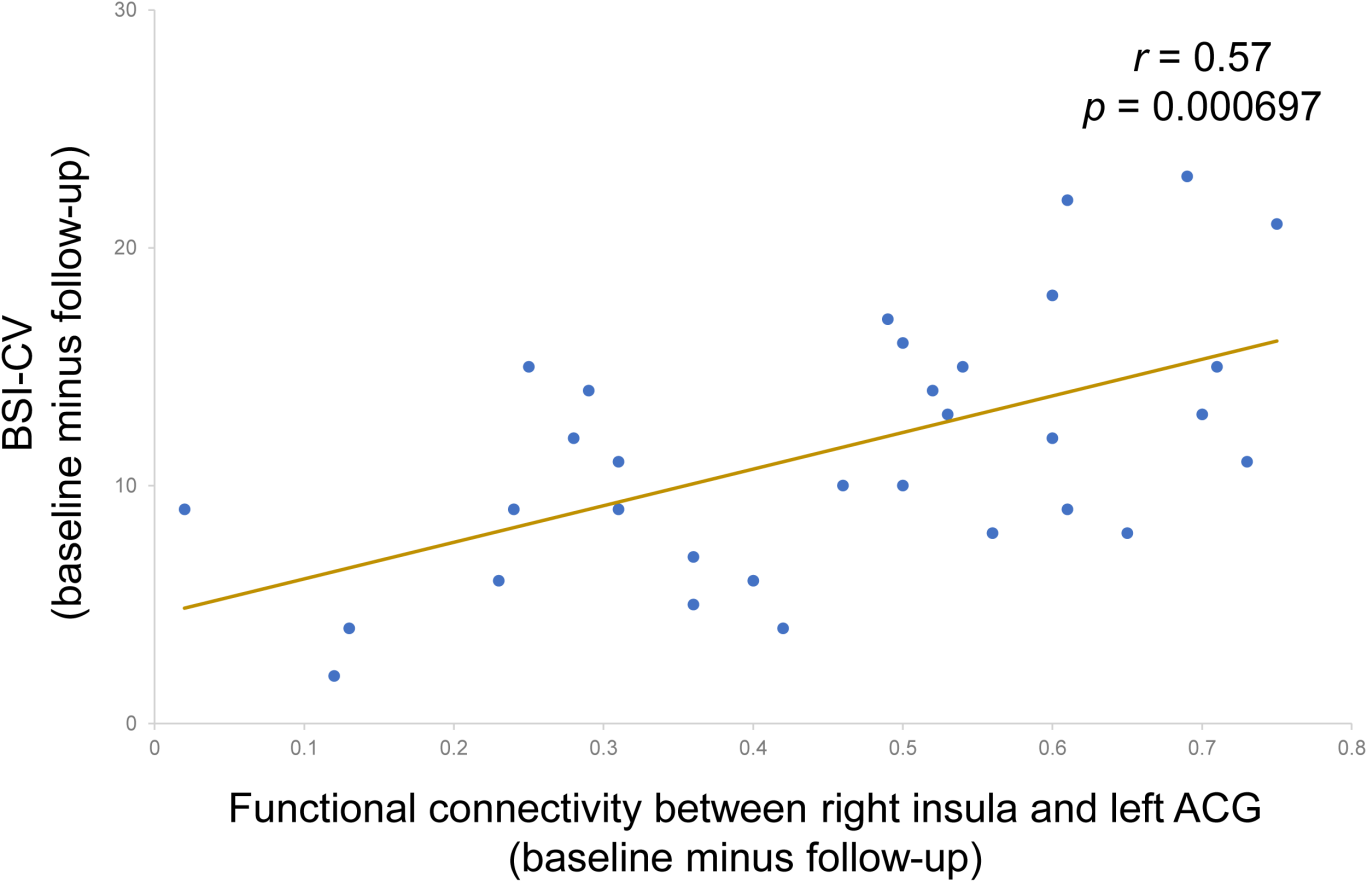
A significant positive correlation between improvements in BSI-CV and changes in functional connectivity between right insula and left ACG (*r* = 0.57, *p* < 0.001). BSI-CV: Beck Scale for Suicide Ideation-Chinese Version. ACG, anterior cingulate gyrus. Follow-up: immediately after the last treatment.

Although no significant correlations were found between the small-world properties and clinical characteristics, a significant positive correlation was found between improvements in BSI-CV (baseline score minus score acquired immediately after the last treatment) and changes in functional connectivity between right insula and left ACG (*r* = 0.57, *p* < 0.001). The correlation result is shown in **Figure 4**.

## Discussion

This study focused on the manifestation of cerebral topological structure in MDD/SI+ patients. Using MRI data based on graph-theory analysis, the functional network of all these three groups was found to conform to small-world topology. However, at baseline, MDD/SI+ patients exhibited impaired and inefficient small-world properties and aberrant functional segregation and functional integration compared with the HCs. These properties showed a renormalization after IT-TMS treatment. We found that patients after IT-TMS treatment targeting individualized DLPFC sites led to significant reduction in suicidal ideation and depressive symptom. The clinical scales in **Table 2** demonstrated the downtrend of BSI-CV and HAMD-17 scores till the end of 30 days after treatment, which confirmed the lasting effects of IT-TMS. Some key nodes were discovered, including superior frontal gyrus, ACG, insula, amygdala, thalamus and middle frontal gyrus (orbital part). Furthermore, decreased functional connectivity between right insula and left ACG correlated with improvement in BSI-CV scores. The current study highlights that MDD/SI+ patients in this cohort showed abnormal brain network connections compared to healthy controls, and that TMS may exert its treatment effects by reducing spontaneous hyper-connectivity in the salience network and insula.

Venlafaxine and duloxetine have quick effects on depressive and suicidal behavior. Considering that patients might conduct suicidal behavior if medications were taken away, they took venlafaxine or duloxetine constantly during IT-TMS treatment, which also met the principles of ethical review and safety requirements. As a result, false-positive results and confounding effects may exist. The sham TMS group and medication-only group are being conducted to exclude false-positive results. In addition, Alina Zaidi et al. concluded that the combination of rTMS with antidepressants significantly reduced depression severity, increasing response and remission rates (26). In consequence, a conclusion that IT-TMS (concurrent medication) exerts better effects can be drawn, but to what extent IT-TMS may interact with medication remains to be explored.

Brain is a complex information system to coordinate multiple regions as a network, hence it is necessary to study the neural mechanisms of MDD/SI+ from a network perspective. Using graph theory-based analysis methods, MDD/SI+ patients demonstrated poorly efficient network properties (σ), reduced functional segregation (Cp and Eloc) and abnormal functional integration metrics (Lp and Eglob) at baseline (**Figure 2**), indicating that the balance between energy cost and communication efficiency had been broken. Functional segregation refers to local network efficiency, and reduced Cp and Eloc indicates that the “speed” of information transfer between adjacent nodes within a network is compromised (27). Functional integration ensures prompt interregional transfer of information, and abnormal functional integration (lower Eglob and higher Lp) indicates that the parallel information transfer in a brain network and communication efficiency are impaired (28). Consistent with these conclusions, this study indicates that MDD/SI+ in this cohort is a dysconnectivity disorder involving multiple neuronal circuits and brain networks, rather than a focal pathology affecting a single or certain regions. Moreover, the graph-theory metrics (Eloc, Cp, Eglob and Lp) were significantly restored after IT-TMS treatment, indicating that both local and global network efficiency were recovered. The rebuilding of information transfer within a network and recovering of interregional transfer of information ensured the rebalance between energy cost and communication efficiency. As a result, depressive symptoms and suicidal ideation were relieved. The previous studies showed that direction of flow of brain activity from left anterior insula to ACC was significantly reversed in MDD patients, hence ACC-based directed signaling patterns are a potential biomarker for MDD (29). This flow was abnormally from ACC to anterior insula, enlightening us to focus on these two brain regions. Under normal circumstances, the anterior insula is the region that belongs to salience network and integrates physical sensations and sends signals to ACC, the region that controls emotions to maintain normal mental state. Our study used TMS to significantly improve suicidal scales and relieve suicidal symptoms among MDD patients with extra suicidal ideation (**Table 2**), and the insula-related functional connectivity was notably decreased (**Figure 3**, **Table 3**), indicating that the ability of insula to block irrelevant information and prevent interference from sensory signals has improved. This was consistent with illustrations that improvement in MDD is strongly associated with functional recovery in the insula and anterior cingulate area. Additionally, the more improved BSI-CV scales showed, the less functional connectivity between right insula and left ACG (**Figure 4**), suggesting that TMS relieved the interference with insula function. As a result, TMS exerted therapeutic effects by reducing the connectivity between right insula and left ACG, which may be potential imaging markers for the diagnosis and treatment of MDD/SI+.

## Limitations and conclusion

To our knowledge, this study is the first to examine graph-theory network alterations after TMS therapy for MDD/SI+ patients. In conclusion, our study concluded that MDD/SI+ was a dysfunction of multiple brain networks, rather than a focal pathology affecting a single or certain regions. IT-TMS exerted better therapeutic effects by reducing the connectivity between right insula and left ACG, which may ultimately inform a clinical protocol for the remission of suicidal ideation and depressive symptoms.

This study has several limitations. Firstly, the sample size is relatively small, which is limited by the number of enrolled subjects, because this is single-center research. This may not reflect the overall characteristics of MDD/SI+ patients. Secondly, the long-lasting (not limited to 30 days) effects of IT-TMS still need to be verified. Finally, the graph-theory results need to be further explained to verify the relationship between MDD/SI+ and network alterations.

## Data Availability

The raw data supporting the conclusions of this article will be made available by the authors, without undue reservation.

## References

[1] Sokero TP Melartin TK Rytsälä HJ Leskelä US Lestelä-Mielonen PS Isometsä ET. Suicidal ideation and attempts among psychiatric patients with major depressive disorder. J Clin Psychiatry. (2003) 64:1094–100. doi: 10.4088/jcp.v64n0916 14628986

[2] Jeon HJ Lee J-Y Lee YM Hong JP Won S-H Cho S-J. Unplanned versus planned suicide attempters, precipitants, methods, and an association with mental disorders in a korea-based community sample. J Affect Disord. (2010) 127:274–80. doi: 10.1016/j.jad.2010.05.027 20591495

[3] Huang Y Wang Y Wang H Liu Z Yu X Yan J. Prevalence of mental disorders in China: A cross-sectional epidemiological study. Lancet Psychiatry. (2019) 6:211–24. doi: 10.1016/s2215-0366(18)30511-x 30792114

[4] Abdelnaim MA Langguth B Deppe M Mohonko A Kreuzer PM Poeppl TB. Anti-suicidal efficacy of repetitive transcranial magnetic stimulation in depressive patients: A retrospective analysis of a large sample. Front Psychiatry. (2020) 10:929. doi: 10.3389/fpsyt.2019.00929 31969842 PMC6960193

[5] Kang S-G Na K-S Choi J-W Kim J-H Son Y-D Lee YJ. Resting-state functional connectivity of the amygdala in suicide attempters with major depressive disorder. Prog Neuropsychopharmacol Biol Psychiatry. (2017) 77:222–7. doi: 10.1016/j.pnpbp.2017.04.029 28445688

[6] Holmes SE Scheinost D Finnema SJ Naganawa M Davis MT DellaGioia N. Lower synaptic density is associated with depression severity and network alterations. Nat Commun. (2019) 10:1529. doi: 10.1038/s41467-019-09562-7 30948709 PMC6449365

[7] Bullmore E Sporns O. Complex brain networks: graph theoretical analysis of structural and functional systems. Nat Rev Neurosci. (2009) 10:186–98. doi: 10.1038/nrn2575 19190637

[8] Chase HW Segreti AM Keller TA Cherkassky VL Just MA Pan LA. Alterations of functional connectivity and intrinsic activity within the cingulate cortex of suicidal ideators. J Affect Disord. (2017) 212:78–85. doi: 10.1016/j.jad.2017.01.013 28157550 PMC5358995

[9] Kim K Kim S-W Myung W Han CE Fava M Mischoulon D. Reduced orbitofrontal-thalamic functional connectivity related to suicidal ideation in patients with major depressive disorder. Sci Rep. (2017) 7:15772. doi: 10.1038/s41598-017-15926-0 29150619 PMC5693996

[10] Mheich A Wendling F Hassan M. Brain network similarity: methods and applications. Netw Neurosci. (2020) 4:507–27. doi: 10.1162/netn_a_00133 PMC7462433 32885113

[11] Sporns O. Graph theory methods: applications in brain networks. Dialogues Clin Neurosci. (2018) 20:111–21. doi: 10.31887/DCNS.2018.20.2/osporns PMC6136126 30250388

[12] Stam CJ Reijneveld JC. Graph theoretical analysis of complex networks in the brain. Nonlinear BioMed Phys. (2007) 1:3. doi: 10.1186/1753-4631-1-3 17908336 PMC1976403

[13] Wagner G Li M Sacchet MD Richard-Devantoy S Turecki G Bär K-J. Functional network alterations differently associated with suicidal ideas and acts in depressed patients: an indirect support to the transition model. Transl Psychiatry. (2021) 11:100. doi: 10.1038/s41398-021-01232-x 33542184 PMC7862288

[14] Sankar A Scheinost D Goldman DA Drachman R Colic L Villa LM. Graph theory analysis of whole brain functional connectivity to assess disturbances associated with suicide attempts in bipolar disorder. Transl Psychiatry. (2022) 12:7. doi: 10.1038/s41398-021-01767-z 35013103 PMC8748935

[15] Wagner G de la Cruz F Köhler S Pereira F Richard-Devantoy S Turecki G. Connectomics-based functional network alterations in both depressed patients with suicidal behavior and healthy relatives of suicide victims. Sci Rep. (2019) 9:14330. doi: 10.1038/s41598-019-50881-y 31586117 PMC6778100

[16] Zhang Y Mu Y Li X Sun C Ma X Li S. Improved interhemispheric functional connectivity in postpartum depression disorder: associations with individual target-transcranial magnetic stimulation treatment effects. Front Psychiatry. (2022) 13:859453. doi: 10.3389/fpsyt.2022.859453 35370853 PMC8964485

[17] Lefaucheur J-P André-Obadia N Antal A Ayache SS Baeken C Benninger DH. Evidence-based guidelines on the therapeutic use of repetitive transcranial magnetic stimulation (Rtms). Clin Neurophysiol. (2014) 125:2150–206. doi: 10.1016/j.clinph.2014.05.021 25034472

[18] Huang Y-Z Rothwell JC. The effect of short-duration bursts of high-frequency, low-intensity transcranial magnetic stimulation on the human motor cortex. Clin Neurophysiol. (2004) 115:1069–75. doi: 10.1016/j.clinph.2003.12.026 15066532

[19] Blumberger DM Vila-Rodriguez F Thorpe KE Feffer K Noda Y Giacobbe P. Effectiveness of theta burst versus high-frequency repetitive transcranial magnetic stimulation in patients with depression (Three-D): A randomised non-inferiority trial. Lancet. (2018) 391:1683–92. doi: 10.1016/s0140-6736(18)30295-2 29726344

[20] Williams N. Stanford accelerated intelligent neuromodulation therapy for suicidal ideation (Saint-si). Biol Pyschiat. (2019) 85:S28. doi: 10.1016/j.biopsych.2019.03.081

[21] Tang Y Jiao X Wang J Zhu T Zhou J Qian Z. Dynamic functional connectivity within the fronto-limbic network induced by intermittent theta-burst stimulation: A pilot study. Front Neurosci. (2019) 13:944. doi: 10.3389/fnins.2019.00944 31572111 PMC6753168

[22] Cole EJ Stimpson KH Bentzley BS Gulser M Cherian K Tischler C. Stanford accelerated intelligent neuromodulation therapy for treatment-resistant depression. Am J Psychiatry. (2020) 177:716–26. doi: 10.1176/appi.ajp.2019.19070720 32252538

[23] Fox MD Buckner RL White MP Greicius MD Pascual-Leone A. Efficacy of transcranial magnetic stimulation targets for depression is related to intrinsic functional connectivity with the subgenual cingulate. Biol Psychiatry. (2012) 72:595–603. doi: 10.1016/j.biopsych.2012.04.028 22658708 PMC4120275

[24] Tang N Sun C Wang Y Li X Liu J Chen Y. Clinical response of major depressive disorder patients with suicidal ideation to individual target-transcranial magnetic stimulation. Front Psychiatry. (2021) 12:768819. doi: 10.3389/fpsyt.2021.768819 34803776 PMC8602581

[25] Chandler J. Brain stimulation law: legal issues raised by current and emerging neuromodulation therapies. Brain Stimulation. (2023) 16:116. doi: 10.1016/j.brs.2023.01.008

[26] Zaidi A Shami R Sewell IJ Cao X Giacobbe P Rabin JS. Antidepressant class and concurrent rtms outcomes in major depressive disorder: A systematic review and meta-analysis. eClinicalMedicine. (2024) 75:102760. doi: 10.1016/j.eclinm.2024.102760 39170936 PMC11338161

[27] Duan J Xia M Womer FY Chang M Yin Z Zhou Q. Dynamic changes of functional segregation and integration in vulnerability and resilience to schizophrenia. Hum Brain Mapp. (2019) 40:2200–11. doi: 10.1002/hbm.24518 PMC6865589 30648317

[28] Yu M Dai Z Tang X Wang X Zhang X Sha W. Convergence and divergence of brain network dysfunction in deficit and non-deficit schizophrenia. Schizophr Bull. (2017) 43:1315–28. doi: 10.1093/schbul/sbx014 PMC5737538 29036672

[29] Mitra A Raichle ME Geoly AD Kratter IH Williams NR. Targeted neurostimulation reverses a spatiotemporal biomarker of treatment-resistant depression. PNAS. (2023) 120:e2218958120. doi: 10.1073/pnas.2218958120 37186863 PMC10214160

